# Supplementary material for: Photobody formation spatially segregates two opposing phytochrome B signaling actions of PIF5 degradation and stabilization
Source: Nat Commun. 2024 Apr 25;15:3519. doi: 10.1038/s41467-024-47790-8 (PMC11045832; doi:10.1038/s41467-024-47790-8)
Supplement: Supplementary file 1 — Supplementary Information [file 41467_2024_47790_MOESM1_ESM.pdf]

**Supplementary Table 1. List of primers used for plasmid construction.**

| Description | Sequence 5'-3' |
| --- | --- |
| *PIF5pro::4xMyc-mCherry-PIF5* construct for generating *mCherry-PIF5/pif5-3* lines | |
| PIF5pro_F | ATTCGAGCTCGGTACCCGGGTTTCTGATAAATTCAATTTGCT |
| PIF5pro_R | TTAATTAACCCTTCCATGTCAGATCTGTAAA |
| 4xMyc_F | TCTGACATGGAAGGGTTAATTAACGGTGAACAAAAG |
| 4xMyc_R | CTTTACTCATGCTACCGTTCAAGTCTTCCT |
| mCherry2L-linker_F | GAACGGTAGCATGAGTAAAGGAGAAGAAAA |
| mCherry2L-linker_R | CTTTACTCATGCTACCGTTCAAGTCTTCCT |
| PIF5_F | TCCCACCCCTATGGAACAAGTGTTTGCTGATTGG |
| PIF5_R | GAAAGCTCTGCATGCCTGCATCAGCCTATTTTACCCATATGAAG |
| *UBQpro::3HA-mCherry-PIF5* construct for generating *mCherry-PIF5/PBC* lines | |
| UBQ10_F | ATTCGAGCTCGGTACCCGGGGTTTTGTGTATCATTCTTGT |
| UBQ10_R | GAACATCGTATGGGTACTGCATCTGTTAATCAGAAA |
| 3HA_F | TACCCATACGATGTTCCTGACTATGCGGGC |
| 3HA_R | CTTCTCCTTTACTAGCGTAATCTGGAACGTCATATGGATATGAGCC |
| mCherry_F | ATATGACGTTCCAGATTACGCTAGTAAAGGAGAAGAAAAC |
| mCherry_R | AGGGGTGGGAGTTGGTGTTGGAGTAGGTTTGTATAGTTCATCCATGCCAC |
| PIF5_F | CAACACCAACTCCCACCCCTATGGAACAAGTGTTTGCTGA |
| PIF5_R | GAAAGCTCTGCATGCCTGCATCAGCCTATTTTACCCATAT |
| *gPHYB-CFP* construct for generating *gPBC* lines | |
| PHYBpro_F | TTACGAATTCGAGCTCGGTACCCAGAGATTATGAGAGAACGAACACTT |
| (linker)-PHYB_R | AGGGGTGGGAGTTGGTGTTGGAGTAGGATATGGCATCATCAGCATCATGT |
| (linker)-CFP_F | CCTACTCCAACACCAACTCCCACCCCTATGGTGAGCAAGGGCGAGGAGCTG |
| CFP-R | TCATACCAACTGAAGTGTGATTACTTGTACAGCTCGTCCATG |
| PHYB_terminator_F | TGGACGAGCTGTACAAGTAATCACACTTCAGTTGGTATGAG |
| PHYB_terminator_R | CGACGGCCAGTGCCAAGCTTGCATGCCTGCAGCAGCCTGGAGAGCATA |

**Supplementary Table 2. List of primers used for qRT-PCR analysis.**

| Accession | Gene name | Forward primer | Reverse primer |
|---|---|---|---|
| AT1G13320 | *PP2A* | TATCGGATGACGATTCTTCGTGCAG | GCTTGGTCGACTATCGGAATGAGAG |
| AT3G59060 | *PIF5* | GCAGCTCCAAGCACAGAACCAAAT | TTGTTGTTGCACGGTCTGCATCTG |
| AT2G18790 | *PHYB* | AAAAGATGTTGTGGAGTGGTTG | TTGATTTCTTTCGCAGTGTGAG |
